# Supplementary material for: Mixed-Precision Ab Initio Tensor Network State Methods Adapted for NVIDIA Blackwell Technology via Emulated FP64 Arithmetic
Source: J Chem Theory Comput. 2026 Apr 20;22(13):6275–82. doi: 10.1021/acs.jctc.6c00203 (PMC13374020; doi:10.1021/acs.jctc.6c00203)
Supplement: Supplementary file 1 [file ct6c00203_si_001.pdf]

Supporting Information for Publication: Mixed-precision ab initio tensor network state methods adapted for NVIDIA Blackwell technology via emulated FP64 arithmetic

Cole Brower,[1,*] Samuel Rodriguez Bernabeu,[1,†] Jeff Hammond,[2,‡] John Gunnels,[1,§] Sotiris S. Xantheas,[3,4,¶] Martin Ganahl,[5,**] Andor Menczer,[6,7,††] and Örs Legeza[6,8,9,10,‡‡]

[1]*NVIDIA, 2788 San Tomas Expressway, Santa Clara, CA 95051*
[2]*NVIDIA Helsinki Oy, Porkkalankatu 1, 00180 Helsinki*
[3]*Advanced Computing, Mathematics, and Data Division, Pacific Northwest National Laboratory, Richland, Washington 99354, USA*
[4]*Department of Chemistry, University of Washington, Seattle, WA 98195, USA*
[5]*SandboxAQ, Palo Alto, California, USA*
[6]*Strongly Correlated Systems Lendület Research Group, Wigner Research Centre for Physics, H-1525, Budapest, Hungary*
[7]*Eötvös Loránd University, Pázmány Péter Sétány 1/C, 1117 Budapest, Hungary*
[8]*Dynaflex LTD, Zrínyi u 7, 1028 Budapest, Hungary*
[9]*Institute for Advanced Study,Technical University of Munich, Germany, Lichtenbergstrasse 2a, 85748 Garching, Germany*
[10]*Parmenides Stiftung, Hindenburgstr. 15, 82343, Pöcking, Germany*

(Dated: March 26, 2026)

In this supporting information we present further numerical results supporting our numerical analysis and conclusions.

In Figs. S1 and S2 we summarize results for systems discussed in Figs. 1 and 2 but obtained on a DGX H100 supercomputer.

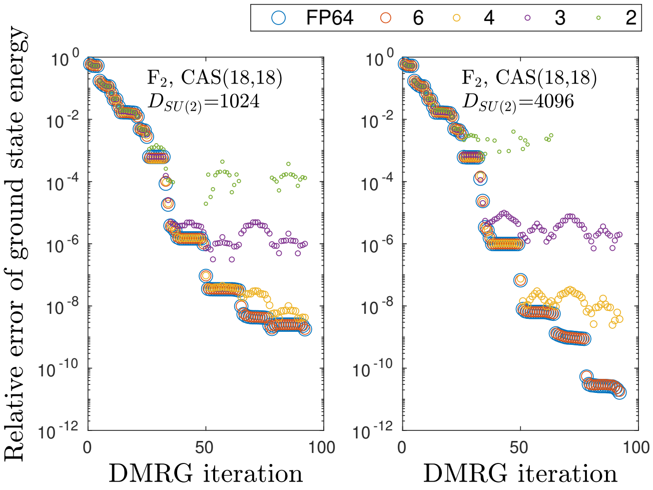

FIG. S1. Similar to Fig. 1 but obtained on a DGX H100 supercomputer.

In Fig. S3 the relative error of the ground state energy, $\Delta E_{\rm rel}$, as a function of DMRG iteration steps is shown for the F_2 molecule in a CAS(18,18) model space using bond dimension $D_{SU(2)} = 1024$, $\kappa = 3$ slices, setting the residual error threshold to $\varepsilon = 10^{-5}$ in the Lánczos diagonalization and by switching between CPU and GPU implementations for the renormalization (network contraction) and SVD algorithmic parts. We observe that employing a CPU or GPU based renormalization procedure, which is mainly based on DGEMM operations, has only minor effect on the obtained convergence profile. In contrast to this, switching from the NVIDIA reduced precision cuSOLVER-based GPU implementation of the SVD step (diagonalization of the reduced density matrix) to the CPU variant the accuracy improves signif-

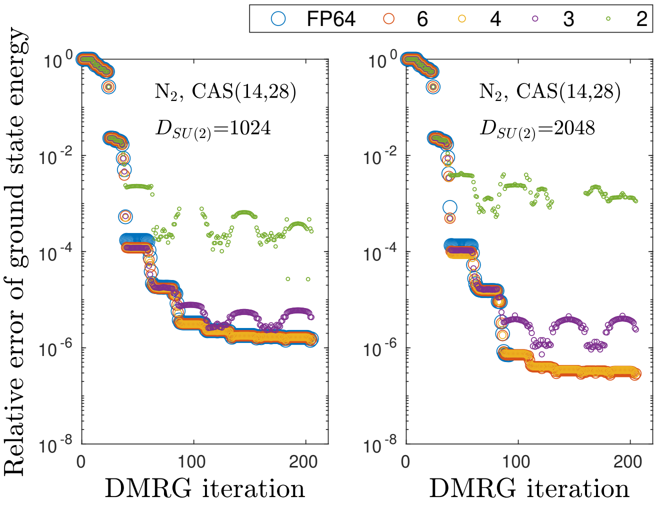

FIG. S2. Similar to Fig. 2 but obtained on a DGX H100 supercomputer.

* cbrower@nvidia.com
† srodriguezbe@nvidia.com
‡ jeffpapers@nvidia.com
§ jgunnels@nvidia.com
¶ Sotiris.Xantheas@pnnl.gov
** martin.ganahl@sandboxaq.com
†† menczer.andor@wigner.hu
‡‡ legeza.ors@wigner.hu

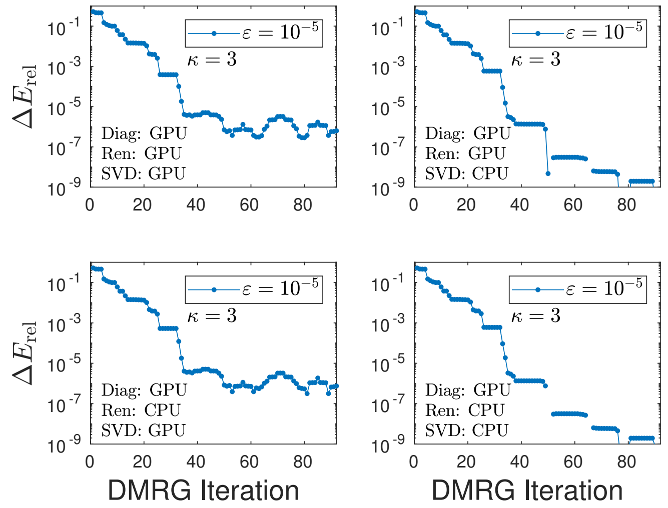

FIG. S3. Relative error of the ground state energy, $\Delta E_{\rm rel}$ as a function of DMRG iteration steps for the F_2 molecule in a CAS(18,18) model space using bond dimension $D_{SU(2)} = 1024$, $\kappa = 3$ slices, setting the residual error threshold in the Lánczos diagonalization method to $\varepsilon = 10^{-5}$ and employing various CPU and GPU implementations for the renormalization (network contraction) and singular value decomposition (SVD).

icantly. Therefore, the error accumulated via SVD using small number of slices, $\kappa = 3$, determines the overall convergence. Note that similar improvement is achieved by using $S \in \{7, 8\}$ slices or native FP64 in cuSOLVER.

In Fig. S4 the relative error of the ground state energy, $\Delta E_{\rm rel}$, as a function of DMRG iteration steps is shown for the F_2 molecule in a CAS(18,18) model space using $D_{SU(2)} = 1024$ and $\kappa = 2$ slices for various residual error threshold values, ε, employed in the Lánczos method. By reducing the residual error, ε, to $10^{-4}, \ldots, 10^{-2}$ the number of non-variational eigenvalues disappeared, leading to an oscillating curve in relative energy in the range of 10^{-4} as in Fig. 1 but without "missing" data points.

In Fig. S5 we present similar analysis as shown in Fig. S3 but for $\kappa = 2$ slices. Similarly, as discussed for Fig. S3, employing a CPU or GPU based renormalization procedure has only minor effect on the obtained convergence profile. In contrast to this, switching from the reduced precision cuSOLVER-based GPU implementation of the SVD step (diagonalization of the reduced density matrix) to the CPU variant the accuracy improves significantly. Therefore, as expected, the large error accumulated via SVD using only $\kappa = 2$ slices prohibits DMRG to converge.

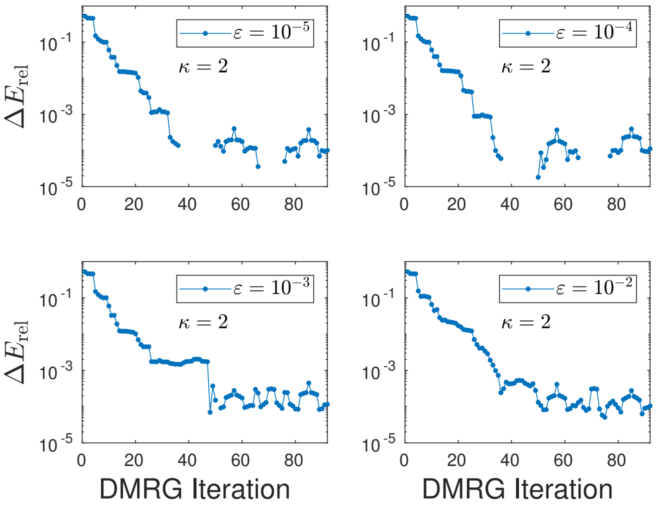

FIG. S4. Relative error of the ground state energy, $\Delta E_{\rm rel}$, as a function of DMRG iteration steps for the F_2 molecule in a CAS(18,18) model space using bond dimension $D_{SU(2)} = 1024$ and $\kappa = 2$ slices for various pre-set residual error threshold values, ε, employed in the Lánczos diagonalization method. Note the missing data points due to non-variational eigenvalues.

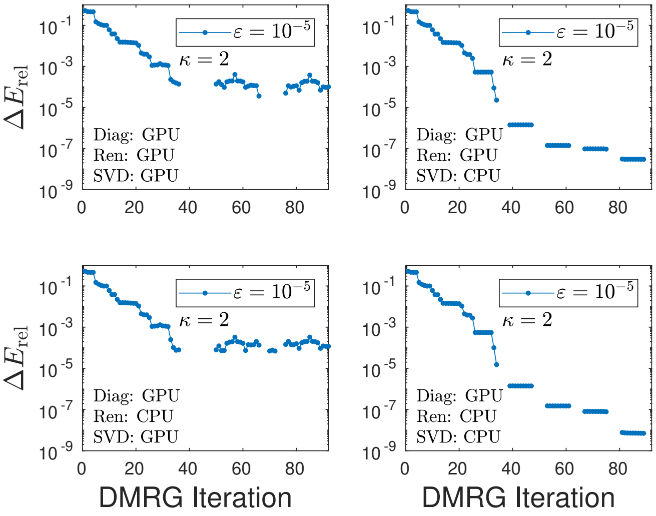

FIG. S5. Similar to Fig S3 but for $\kappa = 2$ slices.
